# ‘*Where is the damned collection*?’ Charles Davies Sherborn’s listing of named natural science collections and its successors

**DOI:** 10.3897/zookeys.550.10073

**Published:** 2016-01-07

**Authors:** Michael A. Taylor

**Affiliations:** 1 School of Museum Studies, University of Leicester & Department of Natural Sciences, National Museums Scotland, Chambers St., Edinburgh EH1 1JF, Scotland (http://orcid.org/0000-0002-1495-8215)

**Keywords:** Charles Davies Sherborn, collections, geology, biology, taxonomy, museums

## Abstract

C. D. Sherborn published in 1940, under the imprint of Cambridge University Press but at his own expense, *Where is the – Collection*? This idiosyncratic listing of named natural science collections, and their fates, was useful, but incomplete, and uneven in its accuracy. It is argued that those defects were inevitable, given Sherborn’s age and wartime conditions, and that what might seem one of Sherborn’s less impressive works was in fact a pioneering work highly influential in stimulating the production of successor works now much used in curation, and in systematic and descriptive biology and palaeontology. The book also contributed to the development of collections research in the natural sciences, and the history of collections and of museums.

## Introduction

**Figure 1.**
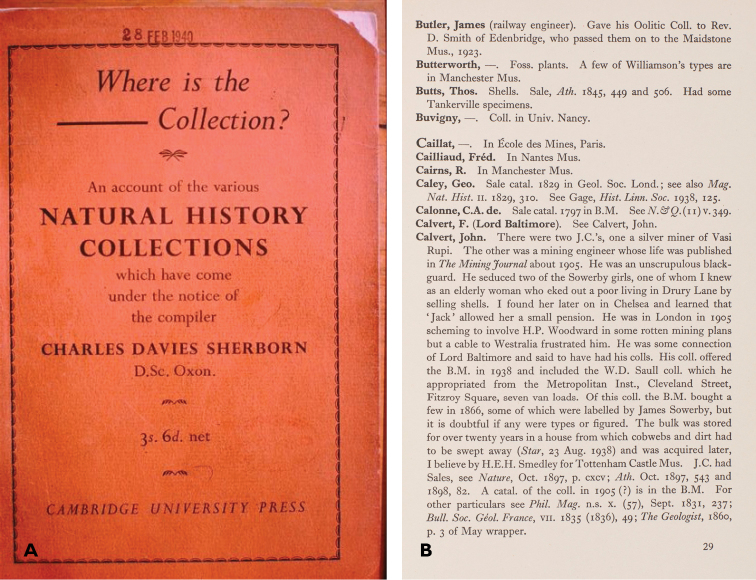
**A** The cover of *Where is the – Collection*? **B** A sample page (p. 29) from *Where is the – Collection*?, including the entry for John Calvert. The Sowerby women are thought to be the daughters of G. B. Sowerby I (1788–1854) (R. J. Cleevely, pers. comm. 2014).

Charles Davies Sherborn (1861–1942) was a geologist and above all a scientific bibliographer (Anonymous 1942b, [Hinton] 1942, Norman 1942, 1944, Griffin 1953, Cleevely 2004, Dickinson 2016, Evenhuis 2016, Shindler 2016, Welter-Schultes et al. 2016). His last significant publication was a small book called *Where is the – Collection*? (Fig. 1a, b; Sherborn 1940). This paper describes the book’s genesis and content, and assesses its significance at the time, the value of its contained information, and its importance as a precedent and nucleus for systematics, curation and collections-historical research in the natural sciences.

Sherborn did not explicitly give his reasons for writing the book. It is evident from his introduction that the aim was to help researchers, and especially systematists, locate named collections, and thereby particular specimens: the important point is that the collections were named. The dash in the title is usually taken as standing for the name of the relevant collection, but Sherborn once privately called his book “*Where is the damned Collection*?” (Norman 1944, p. 81), and one reviewer commented that “The difficulty of discovering the resting place of some important specimen [...] doubtless justifies the ‘blue-pencilled’ word which the author may or may not have hinted at in the title” (Ritchie 1940, p. 80; “blue-pencilled” here means censored as an expletive).

Sherborn’s book was, strictly speaking, not the first listing of collections. Cleevely (1983, p. 9) records sporadic lists of collections published as early as 1812, and notes the presence of collections location data in a listing of geologists in the *Fossilium Catalogus* series (Lambrecht et al. 1938). Sherborn himself noted (p. 5) a prior listing of fossil insect collections, and the listing of some British collections in his own *Catalogue of British fossil Vertebrata* (Woodward and Sherborn 1890).

One of the most important early general works on fossils was Sowerby’s *Mineral Conchology* (Cleevely 1983, pp. 7, 9, 11, 14–16). Sherborn (1935) published a paper that listed all the collectors mentioned by the Sowerbys in this work (1812–1846); he cited the references that helped to identify the 237 collector / collections listed. This was a particularly interesting meta-analysis as 28 of those collectors were women, and often very significant participants, such as Etheldred Benett (1776–1845), contrary to the impression one sometimes gets from the secondary and popular literature of today that Mary Anning was almost the only female collector in this period. Certainly Sherborn’s listing of the collectors who provided material for the Sowerbys must have been an important preliminary stage of compiling *Where is the – Collection*.

However, in its wide scope, *Where is the – Collection*? was for decades unique as a practical reference which listed such information on named natural sciences collections and their fates as he had come across in his decades of work at the British Museum. Sherborn’s interests meant that the emphasis was on palaeontological and malacological collections, mainly in Britain, with a sprinkling of other categories such as mineralogy, ornithology, and botany, and manuscripts. Sherborn also commented on collections which had been destroyed, for instance by fire or flood.

## Methods

In this paper, for space reasons, and because they feature strongly in Sherborn’s book, I use palaeontological collections as my main examples, but in fact similar developments occurred across the entire field of natural science collections. Sherborn’s book was a listing of named collections rather than an institutional directory, so I here use “collection” in the sense of a collection of specimens made by a named person or body, rather than the holding institution as a whole. Admittedly this definition is still ambiguous; for instance, it includes both field and cabinet collectors (cf. Torrens 2006, Lucas and Lucas 2014). Sherborn did not attempt to produce a listing of institutions either directly, or indirectly by indexing, and I therefore do not cover lists of institutions in detail (but do refer to them when relevant). However, Sherborn did include some institutional collections, especially when they had been transferred and dispersed amongst other institutions: effectively, they became collections under the name of the original institutions. The modern equivalents of 1940 values are determined using the Bank of England inflation calculator (URL: http://www.bankofengland.co.uk/education/pages/inflation/calculator/flash/default.aspx, accessed 23 January 2014).

References, archives and repositories: where only pagination is given in a reference, Sherborn’s book (1940) is intended. “British Museum”, in the usual shorthand of Sherborn’s time, here denotes the British Museum (Natural History), London, now the Natural History Museum. Repository abbreviations: BMNH, British Museum (Natural History), now NHM; CUL, Cambridge University Library, West Road, Cambridge CB3 9DR, England; Cambridge University Press, University Printing House, Shaftesbury Road, Cambridge CB2 8BS, England; NHM, Natural History Museum, Cromwell Road, London SW7 5BD, England.

## Origin and content

In a guide to sources for collections research in the first *Newsletter* of the new Geological Curators Group (GCG), Hugh Torrens called Sherborn’s book “the only primary source on collections known to me”, and described it admirably (Torrens 1974, pp. 12–13):

[The book has] 149 pages but every other one is blank to allow annotation. [...] It is scarce only 500 copies having been printed. This is an account of the various Natural History Collections which Sherborn came across between 1880–1939. It is not exhaustive or always accurate but contains an immense amount of information. Furthermore it is often fascinating reading. [...] His biography by J. R. Norman [1944] [...] is also equally entertaining reading. His primary interests were geological and palaeontological so there is a useful [for GCG members] bias towards these collections in his book. [...] it had amazingly to be published at his own expense.

Sherborn said that the book contained “facts accumulated over sixty years in answer to inquiries”, and that its “original MS” had “been on my table at the British Museum (Natural History) and of daily use to the Staff or others” (p. [5]). Norman (1944, 80–81) added that “much of his material was collected from old sale catalogues, biographies, obituary notices, museum guides” and the like. No doubt much of Sherborn’s information came as a by-product to his work on *Index Animalium*, but Sherborn plainly carried out additional research, as shown by his file of MSS notes and clippings (still in the BMNH libraries in the 1970s, R. J. Cleevely 1983, and pers. comm. 2014). Examples are Sherborn’s inquiries for John Phillips’s fossils (see below), and his searches through journals such as *Gentleman’s Magazine* and *Notes and Queries*, of which he bought 143 volumes for the purpose (Norman 1944, p. 81).

Sherborn completed his literature searches around March 1939, and in due course finished his manuscript and sent it to the Museums Association. He later reported the disappointing results to a friend in a letter of 27 December 1939:

... that astute body [the Museums Association] hummed over it for two months, and this tho’ I offered to pay for it, that I sent for the MS. back, [and] sent it on to the Cambridge [University] Press [...] (Norman 1944, p. 81).

Sherborn already had an excellent relationship with the Press, who reportedly called him the “best editor” with whom they had ever worked (Norman 1944, p. 79). On 22 November 1939 he wrote to them, evidently as part of an ongoing discussion (CUP archive, CUL UA Pr.A.S.429):

By the by, you might say if you would undertake the publishing, I to pay cost of printing, to keep say fifty copies and give you the remainder if you pay me say 1/6 on all sold copies. This is only a suggestion as I shall want some publisher on the T[itle]. P[age]. and would rather you than anyone.

Sherborn suggested a price of 3/6 or 4/6 (3/6, three shillings and sixpence in pre-decimal United Kingdom currency, is nominally equivalent to 17.5p today but then worth much more). The Press Syndicate decided at its meeting of 8 December 1939 to “undertake the publication of his proposed catalogue of Natural History References, on commission” (CUL UA Pr.V.82, Syndicate Minutes for 1935–1939). The standard ‘Memorandum of Agreement’, i.e. a printed contract for printing and publishing the book at his expense, survives in the Contract Archive at CUP (K. Thompson, Brand Protection Officer, CUP, pers. comm. 2014), bearing Sherborn’s MSS annotations. Sherborn evidently returned it with a covering letter of 12 December (CUL UA Pr.A.S.429). Amongst other matters, he confirmed a print run of 500 copies of which he was to have 50, and suggested that the *Times Literary Supplement* and Nature were “the only papers likely to be of advertising value of such a book, but I leave it to you”. He specified binding in paper: “I cant afford the cloth. Rest of cash available for you when asked for, do not increase it more than you can help for this is a bit of an effort on my part.”

Sherborn soon reported to his friend in the letter of 27 December 1939 cited above:

[...] Cambridge Press [...] accepted my terms, set it up at once, in ten days the whole proofs went back to Cambridge, and it will be printed and ready by mid-January. Cost me £70, sells at 3/6, 500 copies. So that’s that. (Norman 1944, p. 81).

He would need to sell 80% of all copies to recoup his £70, even ignoring other costs (which apparently included 12.5% commission to CUP specified in the Memorandum). The risk was not trivial as £70 was equivalent to almost £4000 in 2012 values. But to call it ‘a bit of an effort’ surely reflected his habitual economy rather than actual poverty, as he was relatively well off (Norman 1944, but see Shindler 2016). After Sherborn’s death, his estate would be valued at £11,619; his executors later sold the copyright and all rights in the stock of the book to CUP for £5 5s (Anonymous 1942a; *England & Wales, National Probate Calendar (Index of Wills and Administrations), 1858–1966*, probate granted at Llandudno, 5 September 1942; receipt attached to Memorandum of Agreement in CUP Contract Archive).

**Figure 2.**
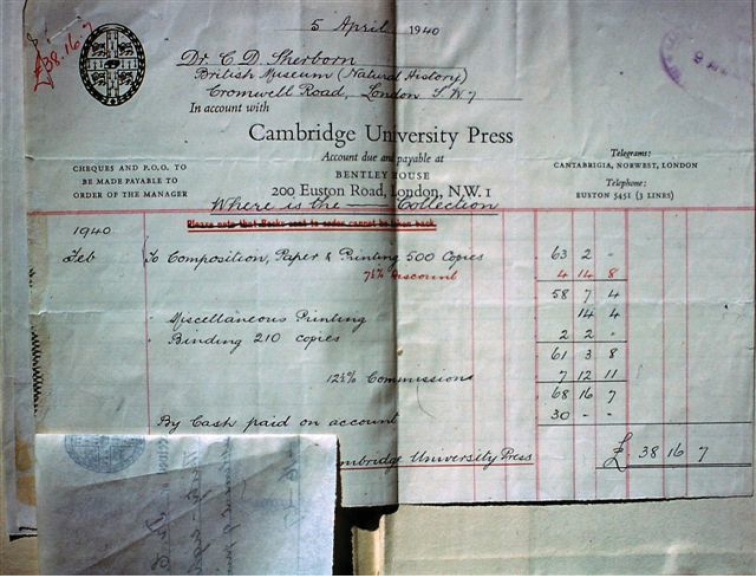
The final bill from Cambridge University Press, tipped into a copy of *Where is the – Collection*? in the NHM Library. Photo courtesy R. J. Cleevely, NHM.

The Press Syndicate Minutes for 2 February 1940 report that the agreement with Sherborn was ‘completed’, whatever that meant (CUL UA Pr.V.82). The printing and binding were in any case done in time for the final bill, dated 5 April 1940, which came to just under the expected £70 (Fig. 2). The book was out in time to be reviewed in the 20 July 1940 issue of *Nature* (Ritchie 1940). An initial search of the CUP archives and of sales catalogues for the period has not turned up any record of an official publication date (R. Grooms, CUP Archivist, CUL, pers. comm. 2014), but this may simply reflect the book’s anomalous nature, the lack of advertising, and the wartime conditions.

Sherborn’s letter of 27 December gives the impression that he withdrew his book from the Museums Association because of the Association’s dilatoriness, but he does not give any reason for this delay. It is possible that the Association had reservations about the book itself, especially if it had the book assessed by the same person who later reviewed it harshly for the Association’s *Museums Journal* (quoted below, “C. M.”, 1940). Another possibility is that the Museums Association feared that some entries were defamatory. Sherborn was seemingly inclined to unrestrained criticism at times. In 1888 the Royal Society of London refused him support for his bibliography of foraminiferans because of his savage review of a foreign rival (Miller 2016, Shindler 2016). In a 1905 letter to Arthur Smith Woodward, then Keeper of Palaeontology, Sherborn described the British Museum’s Chalk echinoderms as “disgraceful material” (NHM Archives DF 100/39/256; P. M. Cooper, pers. comm. 2014). Sherborn was just as forthright in his little book, noting for instance that Professor W. J. Sollas (1849–1936) “destroyed the *Phascolotherium* jaw” of an exceptionally rare Jurassic mammal in the James Parker collection at Oxford (p. 105). The University Museum’s curators have never forgiven Sollas, who was trying to study the jaw’s internal structure through serial sectioning, a crude and inherently destructive predecessor of computed tomography (Vincent 1994, pp. 28–29, 39).

As part of the standard agreement, Sherborn had to indemnify Cambridge University Press for any libel or copyright claims, and in his letter of 12 December (CUL UA Pr.A.S.429) he said, “[...] please read items Groom and Calvert. All parties are long since dead and my remarks are historically valuable and should stand if possible.” Charles Ottley Groom (1839–1894), an impostor who called himself the Prince of Mantua and Monferrat, went by the Scottish lairdly title of Napier of Merchiston (Davenport-Hines 2004). Sherborn described him as a “notorious rogue and thief, tried to kill Thomas Davies [presumably the geologist (1837–1912)] by dropping a boulder upon him from a high ladder in Tennant’s shop in the Strand” (p. 63). And Sherborn’s account of John Calvert (1814–1897), fraudster, traveller, self-proclaimed mining expert, and mineral collector, was the “most notorious entry in his otherwise genial catalogue of collectors” (Fig. 1), stimulating later research by several historians, according to Cooper (2006, pp. 86–87). In fact, Sherborn seems to have attributed to John some of the doings of Albert (1872–1946), John’s also unscrupulous and then still alive grandson - or son: the Calverts were never too clear about this (Birman 1979, Rothwell 2010, Cooper 2006, pp. 85–105). The Press took the precaution of sending the original proofs of those two entries (but nothing else) to Field Roscoe & Co., a London firm of solicitors, on 1 January 1940, and the firm replied the next day, saying that “there is no doubt that the passages in question are defamatory”. But there was no problem if Calvert and Groom were dead, so long as a small change was made to avoid giving the inadvertent imputation of dubious behaviour on the part of one W. G. Ball, who had been selling Calvert’s material on behalf of another firm of solicitors lumbered with it in lieu of a bad debt (CUL, CUP archive, UA Pr.AS.429; Cooper 2006, p. 101). Sherborn was evidently willing to alter his text, for Mr Ball does not appear in the book.

Sherborn’s comments on some museums obviously did not worry the Press, even though English libel law allows corporations to sue. But they might have created a sticking point for the Museums Association, because some of its institutional members were mentioned unflatteringly. He referred to “the way this local museum [Liverpool Museum] has treated types”, Elgin Museum as “a dump of useful stuff uncared for”, and Wilson’s insects “in Perth Mus[eum]. in ‘shocking state’”, and even the Stebbing collection in the British Museum itself where “most of the spirit had evaporated and specimens were practically useless” (pp. 11, 49, 111, 127, 145; his Perth seems to be that in Scotland rather than Australia, from the admittedly incomplete match of other ‘Perth’ entries with Stace et al. 1987).

However, it seems just as likely, if not more so, that the Museums Association’s real problem with the book lay in its timing. Time must have pressed grievously on Sherborn while he sought to publish this last work of any substance. He was, from 1934, unable to work for long periods, and was becoming increasingly aged and unwell, suffering significant deterioration in 1938, and an episode of poor health in the winter of 1938–1939. He now had to cope with the outbreak of a war whose likelihood he had professed not to take seriously (Norman 1944). The British Museum’s staff and facilities were already being diverted to wartime priorities, and Sherborn would know very well from his Great War experiences how severe such disruption could become. If Sherborn and the Cambridge University Press were as efficient as they seem to have been, then the manuscript was presumably with the Museums Association during September and October 1939, give or take a few weeks either way: in other words, the period of final mobilisation, the declaration of war on 3 September, and the first few weeks of war. The Museums Association would have been hugely distracted by the problems which the war posed for its members and itself, and Sherborn’s book would have seemed a very low priority. Perhaps the Association never even got as far as actually considering Sherborn’s proposal.

In hindsight Sherborn was wise to take the initiative by abandoning the Museums Association, and pushing through the book’s rapid publication elsewhere. His sister died in January 1940, he developed heart disease at the end of 1940, and his last years were a time of increasing wartime disruption at both home and the British Museum, especially after the destructive air raids from September 1940 and the closure of the libraries in 1942, the year of his own death (Norman 1944).

## Assessment

Sherborn’s book was uneven, with the biases in subject content already noted. It was organized only by collector, without any indexing by holding institution. It was inadequately edited. The brevity of its sometimes cryptic entries, with inconsistent names and abbreviations for the Royal Scottish Museum, for instance, annoyed the *Nature* reviewer (Ritchie 1940) – surely James Ritchie (1882–1958), Professor of Zoology at the University of Edinburgh, and previously Keeper of Natural History at that same museum. Doughty (1984, p. 160) described the book as “sometimes a little obscure, veiled [...] in the pedant’s sophist[r]y and waggishness”. I am more inclined to ascribe this to the book’s origin as a collection of notes which acted as personal memory-joggers. The entry for Street Museum in Somerset (now the Alfred Gillett Trust) (p. 129) says in part “Nothing of value except the *Ichthyosaurus* (E. I. White 1934). Wallis of Bristol had a pick of specimens and books”. Sherborn obviously knew what this meant, but the reader needs some background knowledge to conclude that, presumably, Errol White (1901–1985), palaeontologist at the British Museum, had made comments to Sherborn about a visit in 1934, and that Dr F. S. Wallis (d. 1979), Director of Bristol City Museum, had helped with a partial dispersal. Sometimes the reader is left tantalised. “Weeks – (formerly Cox). Had the mechanical spider” (p. 141) actually refers to a popular automaton in the museum of mechanical curiosities in Tichborne Street, London, ca. 1803–1835, assembled by a person named Weeks and stemming, at least in part, from the 18^th^ century collection of James Cox (c. 1723–1800) (Coleman et al. 1902, Altick 1978, Smith 2008). And despite recent studies (Hodgkinson 2006, Miller 2016), one is left in the dark as to why the foram worker Fortescue W. Millett (1833–1915) “kept his rare books in the W. C. under the seat” (p. 97). Did Sherborn mean that Millett had an ultra-superior throne carefully integrated into the room’s wood panelling, with convenient bookshelves designed in? Or was this a triple pun on rare, rear (as in backside), and rears (as in English “public school” slang for lavatories)?

The book, as Sherborn himself admitted, was “not exhaustive; that were too much to expect and almost an impossibility” (p. [5]). Nor is the book particularly reliable in detail (Torrens 1974, pp. 12–13, Cleevely 1983, p. 9). Sherborn stated (p. 107) that some of the fossil collection of John Phillips (1800–1874) was stolen and dumped in the River Thames, but this is now known to be an exaggeration of another author’s canard - though he did take the trouble to check the Blackfriars Bridge engineers’ records (Torrens 1975b, Edmonds 1977, Cleevely 1983, p. 231, Nikolaeva and Morgan 2010). The collection of the Wernerian geologist and mining engineer Thomas Weaver (1773–1855), “used to form hard core of a urinal at Bewdley” (p. 141), seems in fact to have been the unwanted residue after sales and donations to museums (Torrens 2004).

There is some evidence that Sherborn simply decided to stop work and go to print, rather than delay any longer, even if it meant cutting corners. He and his friend W. D. Lang (1878–1966) both cited a relatively unusual source for Mary Anning (1799–1847), a Lyme Regis guidebook (p. 9, Fig. 3 here; Brown [1857], Lang 1950, Taylor and Torrens 2014). Yet Sherborn's entry completely fails to mention her numerous and important Liassic vertebrates in the British Museum. It is true that she did not amass much of a personal collection, being a commercial collector who sold her finds, but this cannot be the reason as the same omission recurs in the entry for Thomas Hawkins (1810–1889) (p. 67, Fig. 4 here)(Torrens 1995, Evans 2010). This suggests that Sherborn did not systematically collate his manuscript with the official history of the British Museum’s collections (British Museum (Natural History) 1904–1912), or have it read over by colleagues such as Lang and the palaeoherpetologist W. E. Swinton (1900–1994). Perhaps this was because of wartime disruption. However, the *Museums Journal* reviewer noted other examples of Sherborn’s failure to collate information from other publications, even ones which Sherborn had cited (“C. M.” 1940).

**Figure 3.**
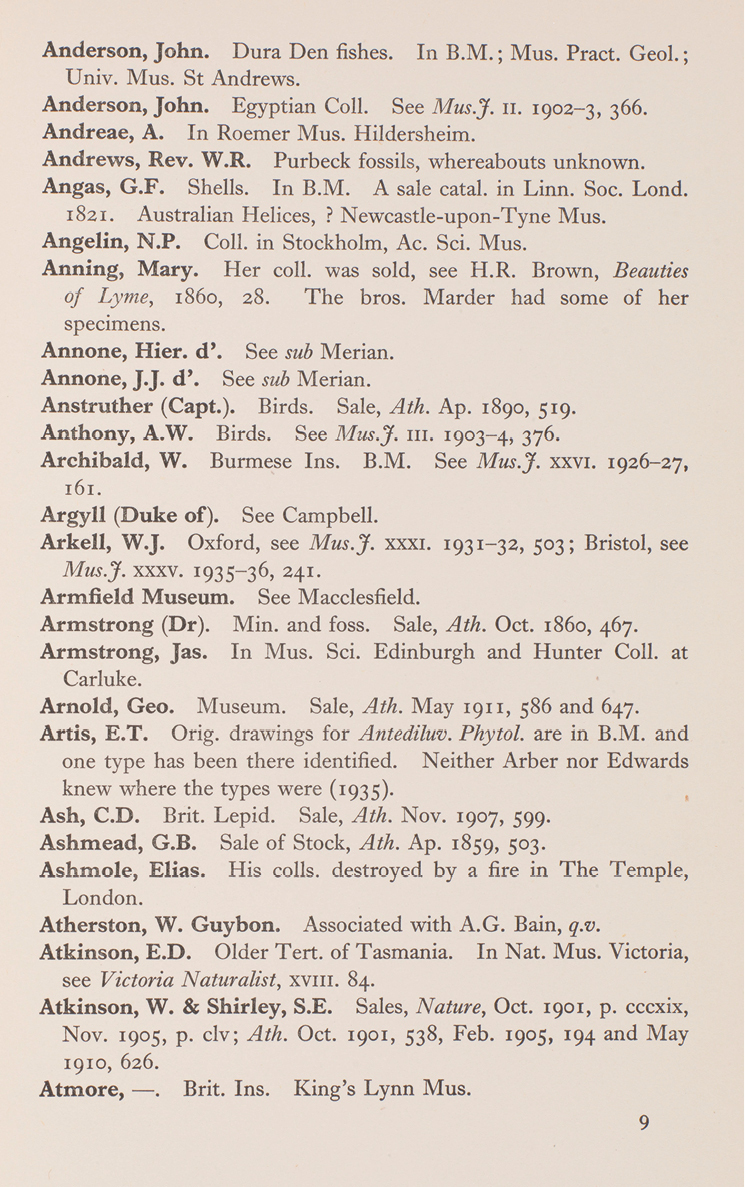
A sample page (p. 9), including the entry for Mary Anning. This misses her specimens in the British Museum (Natural History).

Wartime conditions surely meant that the book received fewer reviews and notices than it might otherwise have had. Even the *Journal of the Society for the Bibliography of Natural History*, co-founded by Sherborn, did not print one till 1943 (Anon. 1943). Reviewers generally noted the book’s incompleteness and, to some extent, unreliability, while focussing on their own areas of expertise. The *Quarterly Review of Biology* reviewed it in 1940 (Anon. 1940), and the ichthyologist George S. Myers (1905–1985) of Stanford University assessed it, sympathetically but briefly, in *Copeia* (Myers 1941). Sherborn’s friend Frederick Chapman (1864–1943), of the National Museum, Melbourne, discussed mainly Australian collections in another sympathetic review in *Victorian Naturalist* (Chapman 1942). A hatchet job in the *Museums Journal* was perhaps written by Claude Morley (1874–1951), an entomologist linked to Ipswich Museum (“C. M.” 1940, p. 73): “the book cannot be described as one which the enquirer may consult with a reasonable probability of finding the whereabouts of any particular collection, even a large one”. There was some truth in this; in *Nature*, Ritchie (1940) noted the omission of important collections in his own Royal Scottish Museum, including that of Hugh Miller (1802–1856) – though, despite Ritchie’s comments, Sherborn was right to mention Miller fossils in Cromarty (Waterston 1954). The CUP archive contains an album with reviews and notices pasted in, presumably recording those known to the Press (CUL, Cambridge University Press newspaper clippings S-1940). Apart from those already mentioned, it contains pieces from *Extraits de la Revue des Questions Scientifiques* (no details, in English); *Science Newsletter*, 17 August 1940; *Biological Abstracts*, Vol. 15, No. 5, 1941; *Ciencia*, Vol. 1, No. 2, 1941 (in Spanish); and *Mexican Society of Natural History*, No. 4, Vol. 1, 1941 (in Spanish).

*Where is the – Collection*? might, at first sight, seem an anticlimactic end to Sherborn’s career, and the least impressive of his works especially when compared to his 11-volume *Index Animalium*. It was, of course, a work of its time. Given Sherborn’s age and the war, he had to publish what he had when he did, or not at all. A separate issue is that for Sherborn to do much better would have involved the organization of a systematic questionnaire, well beyond the energy and resources of a single elderly worker (Dickinson 2016, Evenhuis 2016, Shindler 2016, Welter-Schultes et al. 2016). Moreover, such a questionnaire would have been pointless even if Sherborn could obtain major institutional support. Too many potential target museums were disrupted during the war, even if they did not end up being targets of another kind. Such collections research is, in any case, decentralised by its nature, dealing with collections, documents and archives in many places: far beyond Sherborn on his own in London. It would not help that collections research is naturally more chaotic by nature than Sherborn’s more familiar bibliographical-taxonomic work. A single collection can end up in many places thanks to the vagaries of the owner’s swaps, gifts, sales, and bequests, and then of the holding institutions. For instance, Sherborn (p. 97) failed to note that a significant part of the fossil collection of Charles Moore (1815–1881) at the Royal Literary and Scientific Institution, Bath, had been transferred to Taunton by the honorary curator Rev. H. H. Winwood (1830–1920) (Copp et al. 2000). (Sherborn also stated that the collection at Bath was being cared for by Winwood in 1925: “not an easy task for a man dead 4 years”, dryly noted Torrens 1975a, p. 113).

Some of Sherborn’s information, such as the story of Groom and Davies, plainly came unattributably from colleagues, probably losing precision and introducing error along the way, but with a core of truth, as is the way of oral history. This is perhaps how he knew that A. M. B. Anderson of Brighton was in fact a later alias for Alexander Montagu Browne (1837–1923), curator of the New Walk Museum, Leicester, and a major figure in the history of British taxidermy (p. 7). Rather disappointingly, however, Sherborn failed to confirm the oral tradition amongst successive Leicester curators (including J. A. Cooper and M. Evans, pers. comm. 2014, and MAT) that Montagu Browne was sacked for running a brothel round the corner from the museum; the actual, or at least official, reason was a disagreement with the museum committee over his curatorial training scheme, and perhaps also the museum’s modernisation (McCann 1981, Morris 2010, pp. 339–342). Nevertheless the entry reminds us that Sherborn’s book remains a worthwhile source today, so it is unfortunate that neither the book, nor his biography by Norman (1944), are fully available on the internet today. Might not one of the annotated copies of Sherborn's book at NHM be made available on Biodiversity Heritage Library?

Despite its problems, Sherborn’s book was the only one of its kind, and a great deal better than nothing. Most importantly of all, Sherborn and some (but not all) of his contemporaries appreciated that his book was simply a starting point, an initial stage towards something better, as implied by its publication with every other page left blank. I now turn to the issue of its long–term influence.

## Sherborn’s successors: collections research

A key reason for the rise of the specialist Geological Curators Group (GCG) in Britain and Ireland in the 1970s was the realisation that much needed to be done to improve the quality of museum work in geology (Doughty 1999, Knell 2002). Much of the Group’s attention was devoted to issues of collection care and usage, and specimen conservation.

Survey work was done to find which institutions housed geological material, and the state of these collections and their usage. This work led to publications listing these institutions and analysing the resulting data, notably the classic “State and Status” survey conducted by Phil Doughty (1937–2013) (Doughty 1981, 1999, and more recently Nudds 1999 and Fothergill 2005). Although not collections listings in the Sherborn sense, they often gathered useful information of this kind. Other important examples more globally are Glenister et al. (1977), Prieur (1980), and Webby (1989, 1992).

Under the influence and example of such workers as Hugh Torrens, GCG encouraged research on the history of collections, for it was realised that this had to be understood before a collection could be properly curated and used (Doughty 1984, 1992, 1999, Knell 2002). Such work by Group members and others elucidated, amongst other things, the fates and present locations of collections, and effectively followed on from Sherborn. Some of this research was published as books, such as that which Andrews (1982) wrote specifically to locate certain fossil fish specimens published by Louis Agassiz (1807–1873). But a significant proportion appeared in the *Newsletter of the Geological Curators Group*, latterly titled *The Geological Curator*. This was, and remains, a collective work in progress, with the “Lost and Found” column providing for collections inquiries and for short pieces on new information that does not justify a whole article. This corpus is now available on www.geocurator.org.

Parallel developments took place for biological collections under the aegis of the Biology Curators Group, with its own journals such as the *Biology Curators Group Newsletter*. The Group is now part of the Natural Science Collections Association (NatSCA; an increasing proportion of the older publications are accessible on www.natsca.org).

## Sherborn’s successors: collections reference books

Ron Cleevely of the British Museum became interested in gathering information on collections in the early 1970s, with the intent of producing a new revision of Sherborn’s book, stemming originally from the need to locate type material to support the work of Leslie R. Cox (1897–1965) for the *Treatise of Invertebrate Palaeontology*, and using the data in an annotated copy of Sherborn’s book in the Fossil Mollusca Section. Cleevely developed the book using links with the Society for the Bibliography of Natural History, and with the then new Geological Curators’ Group, including survey data from Doughty’s ‘State and Status’ work and an earlier survey by Douglas Bassett of the National Museum of Wales in 1966–1967 (Anon. 1972, Torrens 1974, Cleevely 1977, 1983, especially introductory essays, Hancock and Pettitt 1981, Pettitt and Hancock 1981, R. J. Cleevely, pers. comm. 2014). Fortunately the Museum’s management recognised the value of this project and Cleevely was able to spend official time on it. He had originally simply intended a more modern version of Sherborn’s effort, and, like it, inexpensive with alternate blank pages. However, its formal adoption by the Museum, and a management decision, led to its publication as a markedly more substantial and more expensive project.

**Figure 4.**
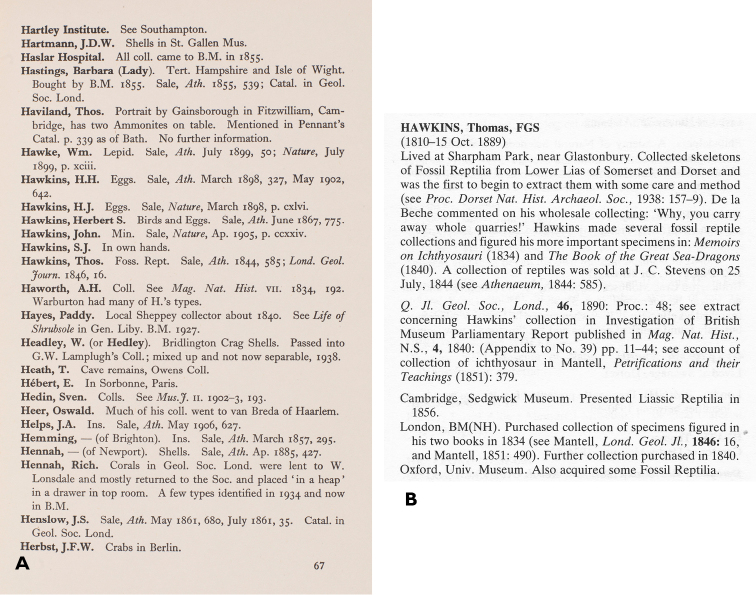
Differing treatments of Thomas Hawkins. **A** The original Sherborn entry in *Where is the – Collection*? (p. 67) **B** The much more extensive entry in *World Palaeontological Collections* (Cleevely 1983, p. 147).

Cleevely’s book *World Palaeontological Collections* provided far more detail than Sherborn, and on many more collections (Cleevely 1983). It systematically incorporated references to collectors’ obituaries and biographies, museum catalogues, and other useful sources (Figure 4). As a result, it is also a very useful biographical reference. It is also much better organised, with indexation by institution and not just collector. Cleevely’s book is not a strict equivalent to Sherborn’s, as he had to restrict his main scope to fossils for reasons of project size (the unused information, mainly on zoological collections, remains on file at NHM). But he did not rigidly exclude minerals, molluscs and other non-fossils if they were relevant, as in the case of a multidisciplinary collector. In origin, spirit and at least partly in coverage, Cleevely’s book is the most direct successor to Sherborn’s. It remains very valuable today, and Doughty (1999) regarded it as notably “worthy of revision”.

Cleevely’s work was preceded, and has been followed by, books listing collections in other natural sciences. Peter Dance’s classic history of shell collecting listed scientifically important collections of Recent shells as an appendix (1966; the 1986 edition appears to have the same appendices though the main text is different). This listing is now largely superseded by Kabat and Boss (1992). Dance (1966: 275, 1986: 201) specifically cited Sherborn’s book as an “extremely useful” predecessor, greatly helping specialists locate important collections. Dance described his own list as containing a fraction of those collections that had existed, and he usually omitted fossil molluscs, but he recorded collections that had been destroyed during the Second World War.

Sherborn’s book listed auction sales, and another direct successor is therefore *Natural history auctions 1700-1972: register of sales in the British Isles* (Chalmers-Hunt et al. 1976). This remains a valuable reference today. “[A]mong bibliographical aids to [its] compilation [...] first and foremost” was Sherborn’s book, both in itself and in an extensively annotated copy in the British Museum (Natural History) (Chalmers-Hunt et al. 1976, pp. ix–x).

Knowing about collections is not just of research value. Area Museum Councils, now mostly abolished in the United Kingdom, were non-governmental public agencies which provided support for, and directed resources to, museums not otherwise funded by central government. During the 1980s, several Area Museum Councils set up advisory schemes to support museums with “orphaned” geological collections, using specialist curatorial and conservation staff, sometimes from larger local museums. This work was in direct response to the depressing results of the GCG’s “State and Status” survey of collections (Doughty 1981, 1999, Taylor 1987, Knell and Taylor 1990). (The same issues, and parallel developments, occurred for biological collections.) An “orphaned” geological collection is one in a museum without specialist geological or natural sciences staff. The persons managing the museum cannot make decisions about the collection, let alone spend resources on it, if they know nothing about it. During the middle decades of the 20^th^ century it was common for a member of staff from a larger institution to remove the scientifically interesting material from an orphan collection, sometimes abandoning or even dumping the remainder. Advisory schemes provided an informed alternative (Knell 1986, Gill and Knell 1988, Knell and Taylor 1990). Non-geological management might still find it hard to understand the scientific importance of their collection, even with specialist advice, but they would have no trouble appreciating its value for public display, and would also grasp the concept of the wider historical and local significance of a collection. However, they needed information and advice to fulfil the collection’s potential. The advisors’ reports, often drawing upon collections research publications such as Cleevely’s book, helped justify expenditure on those collections’ preservation and use. They also raised the regard in which the collections were held, and encouraged the assignment of permanent and temporary staff (e. g. Taylor 1987, Torrens and Taylor 1990, Copp et al. 2000). All those gave geological collections a greater chance to survive in a world where once they had been discarded with impunity.

## Sherborn’s successors: the Collections Research Units, FENSCORE, and other online sources

*Where is the – Collection*? was specifically recognised as a direct predecessor to the Collections Research Units which were organised in the UK during the 1970s and 1980s (Pettitt and Hancock 1981, p. 73). These units stemmed from the push for collections information embodied by the Geological Curators Group and the Biology Curators Group. These Groups’ joint conference with the Systematics Association in Liverpool in 1977, on the “Function of local natural history collections”, led directly to the first scheme, in north-west England, and then to others elsewhere (Hancock and Pettitt 1981, Pettitt and Hancock 1981, Davis and Brewer 1986, Hartley et al. 1987, Stace et al. 1987, especially vi-x, Bateman and McKenna 1993, Museum Documentation Association 1993, Walley 1993, Doughty 1999, Hancock and Hounsome 2010). The Units were usually based in major museums, using the support and regional structure of the Area Museum Councils, and often with additional aid from the government job creation schemes of the time, and from funding bodies such as the Wolfson Foundation. They gathered information on geological and biological collections, mostly in museums and other institutions such as schools and universities, but sometimes held by individuals. Under the influence, in particular, of Bill Pettitt (1937–2009) of Manchester Museum (Hancock and Hounsome 2010), those projects were seen as suitable for computerised data handling. Such modern techniques were also seen as raising the perceived status of natural science curators and helping the survival of their specialist positions. The processed output typically summarised the collector, content, and source localities for each collection, in thick volumes supplemented sometimes by microfiches for the bulk of the detail, as in the Scottish volume (Stace et al. 1987). However, before the entire United Kingdom was covered, these books came to be complemented by an online computer database under the aegis of FENSCORE (Federation for Natural Sciences Collections Recording), founded in 1981 but now dormant. The database is hosted by the University of Manchester (www.fenscore.man.ac.uk). It is understood to contain the data from all regions, including those (such as the South West) for which no book was ever published. It can be searched in different ways from the books. This is a valuable resource, which Doughty (1999) reckoned had basic information on over 95% of the natural sciences contents of museums in the United Kingdom. It does not, however, include the Natural History Museum collections (unless mentioned in some other entry as an “Associated Name”).

This collections research work also fed biological and geological site and locality data into the new county or regional environmental records centres, often based in museums. This work was valuable in itself. It was also useful in gathering political support for those museums which were seen to be responding to the new environmental concerns, and also to be playing their part in job creation schemes at a time when unemployment was a major concern (Ely 1994).

## The future

There seems little immediate prospect of future updates to Sherborn’s successors, or at least those dealing with collections in the United Kingdom. One reason must be the pressure on museum staffing levels, combined with the structural changes within museum organizations which have led to a disproportionate reduction in specialist curatorial staffing over the last two or three decades. All this, combined with the elimination of some Area Museum Councils, inevitably discourages joint curatorial projects, whether between the museums of an area, or by the members of a specialist curatorial group in their own time. Maybe the existing databases are simply seen as sufficiently satisfactory that the further work needed for completion and updating is hard to justify against other pressures and priorities. Perhaps, also, collections research is no longer novel and fashionable, and has to some extent been displaced by newer initiatives relating to such things as social inclusion, health and wellbeing, and communities. New databases seem more likely to be at the specimen rather than collections level, be intended for taxonomic use, and be accessible online. At least initially, too, they seem likely to be at the level of the individual institution, such as the PalaeoSaurus database operated by the British Geological Survey (BGS: http://www.bgs.ac.uk/palaeosaurus/). However, the obvious need for cross-institutional platforms is leading to joint initiatives, if so far still specimen-based ones, such as the JISC-funded and BGS-led *GB3D types online project*, a database of British type fossils, with high-resolution images, stereo-anaglyphs and three-dimensional digital scans (http://www.3d-fossils.ac.uk/home.html). So perhaps we will see the fruition of the early hopes of the Collections Research Units workers for a union catalogue of type specimens (Hancock and Pettitt 1981, Pettitt and Hancock 1981).

Confidentiality has always had to be taken into account (e. g. Bateman and McKenna 1993), but a new problem arises because of legislation (at least in the UK) concerning the confidentiality of personal data on computer databases. This can cause problems where the original collector’s name is part of the data sought by the inquirer. The BGS have had to consider this issue for their databases (M. Howe, pers. comm. 2014). PalaeoSaurus compromises by omitting the donor/collector name from the online display, but one can still search by using the collector name; and more recently donors have been asked to give permission for their names to be put online. In my view, there seems a strong argument for the default position to be the routine publication of names, with them being taken down if requested. The names of collectors and donors can be critical for research, and requests not to publish names are rare or non-existent, while names were routinely published in print in the days when museums still produced full annual reports.

As far as the field as a whole is concerned, one obvious way forward would be a regularly updated digital version of Cleevely’s book, and its equivalents for other fields, perhaps online and presumably incorporating information from FENSCORE. Until then, it seems likely that as far as overall databases are concerned, we will have to rely on Sherborn’s first-generation successors, not forgetting Sherborn himself, and (for the UK) FENSCORE, with internet and literature searches to catch more recent publications. FENSCORE, at least, might perhaps be modernised by converting the data into a modern system of data management, which could be updated directly by curators allowed password access (G. Hancock, pers. comm. 2014). This reminds us of the increasing importance of on-line sources, of which an example is the web publication *2,400 Years of Malacology* by Eugene V. Coan, Alan R. Kabat and Richard E. Petit (http://www.malacological.org/2004_malacology.html). It lists papers about malacologists, such as biographies, bibliographies, and lists of taxa and their present status, often noting the present repositories of relevant type specimens. Most importantly, like other on-line resources, this can be relatively easily updated, as happens near the beginning of each calendar year (E. Coan, pers. comm. 2014).

Some museums also contain historical accounts and other information on their websites, but those sites have a primary role in marketing, education and public presentation, and are liable to radical modification thanks to marketing-driven changes. It is prudent to keep such academic information in an explicitly permanent area, perhaps best of all in a completely separate formal repository.

## Conclusions

Guides such as Sherborn’s are needed more than ever, with the great increase in our knowledge of collections and their fates, and their usage in research and education. See, for instance, the essays by Cleevely (1983) and Doughty (1984, 1992), and the comments above on orphaned collections. Here are, briefly, a few further case studies.

A biologist or palaeontologist may only be concerned with individual specimens of a single taxon, and which institution holds them. But to find those specimens needs a knowledge of collections, the intermediate level between specimen and museum, and also how to use evidence such as specimen labels and catalogues. Such work led to the location of the lost holotype of the ammonite *Ammonites defossus* Simpson, 1843, at the Sedgwick Museum, Cambridge, informing a decision of the International Commission of Zoological Nomenclature (Torrens 1979, Forbes 1980, Brunton et al. 1985).

It can be important to find the institutions holding a named collection. A researcher on the Wealden fossil reptiles of the dinosaur pioneer Gideon Mantell (1790–1852) could find it valuable to know the museums to which his collection was partly dispersed by the British Museum in the late 1880s (Cleevely and Chapman 1992, p. 354).

There are other reasons to be aware of collections as entities in their own right. The documentation of collections, in the widest sense, includes diaries, field notes and correspondence. When a collection is split between museums, one institution is likely to end up holding data relevant to specimens in another institution. An example is the Alfred Leeds (1847–1917) collection of Middle Jurassic fossil vertebrates from Peterborough, England, divided between museums in different countries (Liston and Noè 2004, Araújo et al. 2008, Noè et al. 2010). A knowledge of the collection in question can suggest other important issues; for instance, a researcher using the Jurassic marine reptiles collected by Thomas Hawkins (1810–1889) needs to know that these contain a number of deceptively fabricated composites (Lomax and Massare 2012).

Finally, the creation and use of collections is a major subject of research in its own right, which addresses important questions in the sociology and history of science, and in wider Western culture. A good example is the work of Simon Knell (2000) on early 19^th^ century English geology, which was inspired by Hugh Torrens's biographical studies of an underclass of practical men and women. Torrens (1995) has argued that historians must remember that making a collection can itself be a major contribution to a field of study, even if the collector produces no publications (see also Doughty 1992, pp. 518–519). Such a person was Mary Anning (1799–1847), commercial collector of Lyme Regis. Remarkably, she has attracted more biographical attention than almost all British or Irish geologists (Oldroyd 2013). This admittedly arises partly because of her story as a poor working class woman in a romantic Regency resort, but the excitement of fossil collecting is an important element in her appeal: hence the stream of popular Anningian books and articles, and museum activities such as those at the Natural History Museum, London, and Lyme Regis Museum. All this is, of course, based in part on Torrens’s research (1995), reminding us that collections research has an important role in public education and recreation. Anning is admittedly an ironic example, for we would probably know more about her if Sherborn had not dispersed, and, one presumes, also partly destroyed much of her personal archive as valueless for scientific research. This came about because her papers had been handed to Richard Owen (1804–1892), latterly Director of the British Museum (Natural History), and thereby passed to Sherborn who was given the huge and problematical task of dealing with Owen’s papers (Gruber and Thackray 1992, Torrens 1995). This explains what happened when the American palaeontologist George Gaylord Simpson (1902–1984), visiting the British Museum in 1926–1927 to work on Mesozoic mammals, was befriended by Sherborn. Sherborn gave him “some treasures, an ms & autograph letter of Owen’s, [and] a sheaf of notes in Clift’s hand on the famous ‘Missourium’” – William Clift (1775–1849) being Owen’s father-in-law and predecessor as Conservator at the Hunterian Museum (Laporte 1987, p. 62). This attitude of Sherborn’s must have contributed to the historiographic problems which today beset any writer trying to make sense of what has been written about Anning while paying due respect to elementary accuracy at any level (Torrens 1995, Taylor and Torrens 2014 and refs therein).

A knowledge of collections is, in short, useful for curation and research, and in developing the managerial and political will to support those collections and their museums. But this requires the underpinning of a corpus of organised information about the collections, and this is what Sherborn pioneered, as *Nature*’s reviewer instantly realised (Ritchie 1940, p. 80):

[The book’s] deficiencies can be put right in time; the chief concern is that Dr. Sherborn’s vast knowledge and painstaking labour have created a foundation upon which a complete *Catalogus Thesaurorum* [i.e. Catalogue of Collections] may be erected, and which in the meantime will be invaluable for reference.

For its defects, Sherborn’s book was more than useful enough to show the value of such works, while its inadequacies repeatedly reminded the user that something better was not only possible, but must be done. The seed which he planted did indeed take root and grow. How it will develop in the future is, perhaps, another matter.
